# Differences in the Bacteriome of Smokeless Tobacco Products with Different Oral Carcinogenicity: Compositional and Predicted Functional Analysis

**DOI:** 10.3390/genes8040106

**Published:** 2017-03-23

**Authors:** Nezar Noor Al-hebshi, Fahd Ali Alharbi, Mohammed Mahri, Tsute Chen

**Affiliations:** 1 Department of Maxillofacial Surgery and Diagnostic Sciences, College of Dentistry, Jazan University, 45142 Jazan, Saudi Arabia; dr.m.mahri@hotmail.com; 2 Kornberg School of Dentistry, Temple University, 3223 N Board Street, Philadelphia, PA 19140, USA; 3 Otolaryngology—Head and Neck Surgery Department, Faculty of Medicine, Jazan University, 45142 Jazan, Saudi Arabia; fahdalharbi3@gmail.com; 4 Department of Microbiology, Forsyth Institute, Cambridge, MA 02142, USA; tchen@forsyth.org

**Keywords:** bacteria, bacteriome, carcinoma, microbiome, mouth, smokeless, snuff, tobacco

## Abstract

Smokeless tobacco (ST) products vary significantly in their oral carcinogenicity. Much is known about the differences in the chemical, but not the bacterial, constituents of these products. In this study, we explored the composition and function of the bacteriome in ST products from four countries using quantitative polymerase chain reaction (qPCR) and 16S rRNA-based next generation sequencing. The bacterial load (16S rRNA copies/gram) was lowest in Swedish snus (3.4 × 10^6^) and highest in Yemeni shammah (6.6 × 10^11^). A total of 491 species-level taxa, many of which are potentially novel, belonging to 178 genera and 11 phyla were identified. Species richness and diversity were highest for Swedish snus and lowest for Yemeni shammah. *Bacillus*, *Paenibacillus*, and *Oceanobacillus* spp. were the most abundant in American snuff; species of *Pseudomonas*, *Massilia*, *Propionibacterium*, *Puniceispirillum*, and *Gloeothece* predominated in Swedish snus. In Sudanese toombak, *Facklamia*, *Desemzia*, *Atopostipes*, and *Lysinibacillus* spp. accounted for the majority of the bacteriome. Yemeni shammah exclusively contained *Bacillus* spp. Functional prediction by phylogenetic investigation of communities by reconstruction of unobserved states (PICRUSt) showed that genes encoding cadmium/zinc and nickel transport systems were enriched in the presumptively “high carcinogenicity” products. The bacteriome of ST products thus differed qualitatively, quantitatively, and functionally. The relevance of these differences, particularly with respect to nickel and cadmium, to oral carcinogenesis warrants further investigation.

## 1. Introduction

Smokeless tobacco (ST) refers to forms of tobacco products that are used without burning. They are usually chewed, sucked, or applied to the gingiva, while fine-powdered products are sometimes sniffed through the nose. ST is available in many forms that are used by populations across five continents. According to the International Agency for Research on Cancer (IARC) Working Group on the Evaluation of Carcinogenic Risks to Humans, there is sufficient evidence to support carcinogenicity of ST in humans and to consider it as a cause of cancers of the oral cavity, esophagus, and pancreas [1].

This position, however, is not unanimous, since different ST products seem to significantly vary with respect to their carcinogenicity. For example, overall evidence strongly indicates that use of Swedish snus poses a very small risk of oral cancer development and that use of American chewing tobacco and moist snuff are associated with a very low risk [2,3,4]. On the contrary, strong association has been reported from other parts of the world between oral cancers and use of certain types of ST, including toombak and saffa in Sudan [5,6,7], shammah in Yemen and the South of Saudi Arabia [8,9,10], and almost all forms of chewing tobacco in India [11,12]. This variation in carcinogenicity of ST products has been mainly attributed to differences in the concentrations of carcinogenic chemicals, primarily tobacco-specific *N*-nitrosamines (TSNAs). Swedish snus and contemporary Americans products, for example, have much lower concentrations of TSNAs and other carcinogens compared to Sudanese toombak and Indian products [1].

What has not been explored adequately, despite being believed to have an important role in accounting for differences in the carcinogenicity of ST products, is their microbial content. Bacteria associated with tobacco are known to reduce nitrate into nitrite, which in turn reacts with tobacco alkaloids to form TSNAs [13]; i.e., bacteria are determinants of TSNAs levels in tobacco. In addition, *Bacillus* species recovered from chewing tobacco have been shown to experimentally induce exudation from oral mucosa [14], suggesting that bacteria in ST products may also directly contribute to development of oral cancer by inducing chronic inflammation [15]. However, literature on the microbiology of ST products is sparse. Early reports, mostly by investigators of the tobacco industry, performed identification and quantification of bacteria and fungi in fresh and processed tobacco using cultivation-based methods [15]. Recently, 16S rRNA-based techniques including random fragment length polymorphism (RFLP), denaturing gradient gel electrophoresis (DGGE), single strand conformation polymorphism (SSCP), and sequencing have been used to characterize bacterial communities in fresh and cured tobacco leaves as well as those associated with tobacco fermentation process [16,17,18,19,20]. These studies revealed great deal of diversity and differences in the composition of microbiota associated with the different forms of tobacco. One of them also demonstrated a correlation between the microbial composition of tobacco and its content of TSNAs [20].

Cultivation-independent assessment of bacterial constituents of ready-to use ST products is limited to one very recent study in which next generation sequencing (NGS) with Ion Torrent PGM’s chemistry was employed to profile bacteria in American moist and dry snuff products as well as Sudanese toombak [21]. Samples of Swedish snus were initially also included; however, amplifiable amounts of DNA could not be recovered from them in that particular study. In addition, the description of the results was limited to the family level, which is probably a reflection of the low taxonomic resolution provided by the V4 hypervariable region targeted [22] as well as the analysis pipeline used (a Bayesian classifier using Greengenes 13_5 sequences and taxonomy as reference). In the current study, we characterize the species composition and predict the functional attributes of the bacterial community in ST products with different carcinogenicity, namely samples of American moist snuff, Swedish snus, Sudanese toombak, and Yemeni shammah.

## 2. Materials and Methods

### 2.1. Smokeless Tobacco Products—DNA Extraction

Eleven, ready-to-use ST products were included in the study as follows: four brands of American moist snuff (coded as A1–A4), three brands of Swedish snus (coded as S1–S3), a sample of Sudanese toombak (SuT), and samples of three types of Yemeni shammah, namely black, yellow, and green shammah (BS, YS, and GS, respectively). The American and Swedish products were bought from tobacco shops in New York, NY, USA, and Bergen, Norway, respectively. The Sudanese toombak and Yemeni shammah were obtained from the local market in Khartoum, Sudan, and Gizan, Saudi Arabia, respectively. The samples were stored at room temperature and DNA extraction was performed within three months of purchase, and, in the case of American and Swedish product, before the expiration dates.

A half gram of each product was suspended by vortexing for 10 s at full speed (3300 rpm) in 2 mL Tris EDTA (TE) buffer to recover bacterial cells and then briefly spun at 200 g to precipitate solid matter. Five-hundred microliters of the supernatant, as well as of a negative extraction control, were used for DNA extraction, which involved an initial bead beating step followed by automated extraction on a Maxwell^®^ 16 Research Instrument (Promega, Madison, WI, USA) using the Maxwell 16 Tissue DNA Kit (Promega) according to the manufacturer’s instructions. DNA concentration was measured using a Qubit assay (Life Technologies, St. Louis, MO, USA).

### 2.2. Determination of Bacterial Load

A quantitative polymerase chain reaction (qPCR) assay was performed to determine bacterial load in the extracts. Each reaction was set up to include 5 µL 2X SYBR Green/AmpliTaq Gold DNA Polymerase mix (Applied Biosystems, Foster City, CA, USA), 4 µL DNA template (or negative extraction control) and 1 µL the universal bacterial/archaeal 16S ribosomal ribonucleic acid (rRNA) gene primer set 1406F/1525R [23] (0.4 µM). For each sample, three dilutions were run in triplicate. To control for inhibition, another set of reactions spiked with *Escherichia coli* DH10B genomic DNA was run in parallel using the *E. coli*-specific, *rpsL* primer set [23] (0.2 µM). A standard curve was generated by running 10-fold serial dilutions of *E. coli* DH10B genomic DNA. Amplification was carried on a ViiA7 platform (Applied Biosystems) including an initial enzyme activation cycle at 95 °C for of 10 min followed by 40 cycles of denaturation at 95 °C for 15 s, annealing at 55 °C for 20 s and extension at 72 °C for 30 s. The cycle threshold (Ct) values were recorded and analyzed using ViiA7 v1.2 software. Bacterial load was calculated as 16S rRNA gene copies per 1 gm tobacco sample.

### 2.3. Amplicon Library Preparation and Sequencing

Library preparation and sequencing were done at the Australian Centre for Ecogenomics according to the workflow outlined by Illumina [24] with the exception of replacing the polymerase specified with the Q5 Hot Start High-Fidelity 2X MasterMix (New England Biolabs, Ipswich, MA, USA). Briefly, the degenerate primers 27FYM (AGAGTTTGATYMTGGCTCAG) [25] and 519R (GWATTACCGCGGCKGCTG) [26], modified to contain Illumina’s specific adapter sequences 803F and 1392wR, were used to amplify the V1-3 region of the 16S rRNA gene using standard PCR conditions. The resultant PCR amplicons (≈520 bp) were then purified (Agencourt AMPure XP beads, Beckman Coulter, Brea, CA, USA), indexed with unique 8-base barcodes (Nextera XT v2 Index Kit sets A-D (Illumina, San Diego, CA., USA)) and pooled together in equimolar concentrations. A set of negative amplification controls (mastermix alone and with other reaction components) were included for both the amplicon production and indexing reactions. Finally, sequencing of the indexed library was performed as part of a pool of 192 samples employing the v3 2 × 300 bp chemistry on a MiSeq platform (Illumina, USA) according to the manufacturer’s protocol (targeted depth of 100,000 reads pre sample).

### 2.4. Preprocessing of Sequencing Data

The raw data were submitted to Sequence Reads Archive (SRA) under project no PRJNA339213. Reads with mismatches in the primer sequences were filtered out before the latter were trimmed off. The software PEAR [27] was then employed to stitch paired sequences using the following parameters: minimum amplicon length = 432 bp; maximum amplicon lengths = 522 bp; and *P* = 0.001 (a lower *P* value reduces false positive rates but decreases read merging rates; program default is 0.01). The merged reads were subsequently preprocessed using the mothur software package version 1.38.1 [28]. Firstly, to stringently minimize sequencing errors, reads with ambiguous bases, with homopolymers >8 bases long or that did not achieve a sliding 50-nucleotide Q-score average of ≥35 were filtered out. Secondly, the high quality reads were aligned using Needleman’s method to SILVA reference alignment [29], and those with bad alignment (reads with start and end positions different than those of the majority of the reads) were removed. Finally, the remaining reads were cleared of chimeras with Uchime [30] using the self-reference approach, in which each read is checked against reads with higher abundance in the same sample [31].

### 2.5. Taxonomy Assignment Algorithm

**Figure 1:**
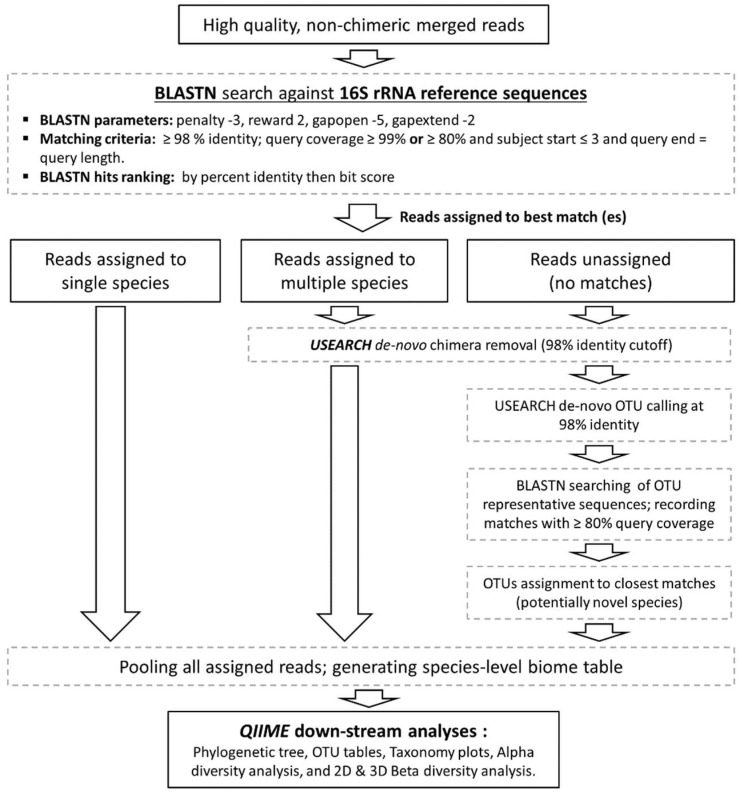
Reads taxonomy assignment. A BLASTN-based algorithm used to classify the reads to the species level and perform down-stream Biological Observation Matrix (BIOM) analysis.

The high quality, non-chimeric reads were classified to the species level employing the BLASTN-based algorithm illustrated in Figure 1. Briefly, reads were individually BLASTN-searched against NCBI’s Microbial 16S rRNA gene reference sequence set (ftp://ftp.ncbi.nlm.nih.gov/blast/db/16SMicrobial.tar.gz) supplemented with a modified version of the Greengenes Gold set (modified-GGG) [32] and the Human Oral Microbiome Database (HOMD) version 14.5 [33]. These combined contain 22,002 well-curated, near full-length reference sequences representing a total of 13,164 microbial species. NCBI’s BLAST version 2.2.28+ was run using the parameters and matching criteria shown in Figure 1, ranking hits by percent identity and, when equal, by bit score. Reads were then classified to the species level based on taxonomy of the top hit reference sequence. If a read returned top hits representing multiple species (two in most cases), it was subject to secondary de novo chimera checking with USEARCH program version v8.1.1861 [34] using a percent identity cutoff of 98% and if found to be non-chimeric, was considered valid and assigned a multiple-species taxonomy. Reads with no matches at the specified criteria were pooled together and subject to the de novo chimera checking as above, and then to species-level de novo operational taxonomy unit (OTU) calling at 98% identity cutoff using USEARCH. The resultant OTUs were labelled as potentially novel species and a representative read from each was BLASTN-searched against the same reference sequence set again to determine the closest species for taxonomy assignment.

### 2.6. Down-Stream Biological Observation Matrix Analysis

All assigned reads (unique species, multispecies, and potentially novel species) were used to generate species-level Biological Observation Matrix (BIOM) table for down-stream analysis with QIIME (Quantitative Insights Into Microbial Ecology) software package version 1.9.1 [35]. The full BIOM table was used to generate taxonomy plots from the species to kingdom level. The samples were then randomly subsampled to obtain equal number of reads across samples, based on the sample with lowest read count (rarefaction). The rarefied BIOM table was subsequently used to calculate species richness and a range of alpha and beta diversity indices. The phylogenetic tree required for constructing the UniFrac-based matrices used in some of the beta diversity analyses, was built dynamically from reference sequences with matched reads.

### 2.7. Imputed Functional Predictions

Mothur was employed to reclassify the sequences using Wang’s method [36] and Greengenes 97% OTUs (version 13.5) as reference, assign them to OTUs based on their taxonomy and generate a BIOM table. Subsequently, PICRUSt (phylogenetic investigation of communities by reconstruction of unobserved states) [37] was used to normalize the OTU table for 16S rRNA copy number variations and then impute the functional bacterial content of each of the samples based on KEGG orthologs (KO) and pathways. Based on evidence from the literature on their oral carcinogenicity, the Swedish and American products were grouped together as “low carcinogenicity” while the Yemeni and Sudanese as “high carcinogenicity.” Differences in genes and pathways between the two groups were explored using Linear Discriminant Analysis Effect Size (LEfSe) [38]. Secondarily, the taxonomy obtained by Wang’s method (Bayesian classifier) was compared with that obtained by the BLASTN-based classification pipeline described above.

## 3. Results

### 3.1. Bacterial Load of the ST Products

**Figure 2:**
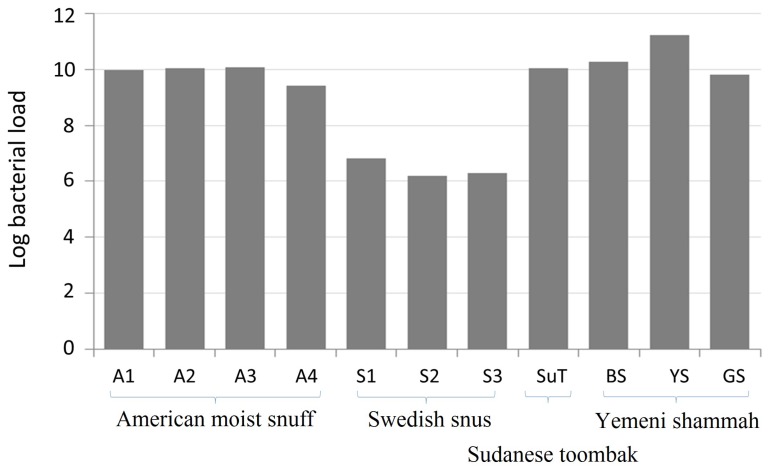
Bacterial load of the smokeless tobacco (ST) products expressed as log_10_-transformed 16S rRNA gene counts per gram. Quantification was performed using quantitative polymerase chain reaction (qPCR) in triplicate.

The log_10_-transformed, absolute bacterial 16S rRNA gene copy counts in each of the samples are presented in Figure 2. The American, Sudanese and Yemeni samples, except YS, had comparable bacterial load at around 10^10^ gene copies per gram. The load was higher by one log in YS (1.7 × 10^11^) and lower by four logs in Swedish snus (3.4 × 10^6^). No amplification was observed for the negative extraction control.

### 3.2. Sequencing and Data Processing Statistics

A total of 1,142,994 raw paired reads were obtained; the negative PCR controls did not generate any background noise. Filtering out reads with primer mismatches removed ≈15% of the sequences. Around 97% of the remaining reads could be successfully stitched with PEAR. At the stringent quality filtration step, 93% of the merged reads were removed. Subsequent alignment and chimera check removed a further 1.3%, leaving a final of 54,970 high-quality, non-chimeric merged reads with an average length of 489 bp. Applying the same read merging and quality-filtration algorithm to data obtained from a mock community in a previous study [39] resulted in 10 fold reduction in sequencing error rate (data not shown).

During the taxonomy assignment stage, 2044 reads were identified as chimeras; the rest were successfully classified to the species level. The final read count per sample ranged from 2265 for SuT to 7955 for S2 (mean of 4811 ± 1738 reads per sample). The results presented below were obtained using a minimum read count per species (MC) of one. Results for higher MC cutoffs (2, 5, 10, 50, and 100) can be found at ftp://www.homd.org/publication_data/20170315/qiime_results/index.html.

### 3.3. Species Richness, Diversity, and Coverage

**Figure 3:**
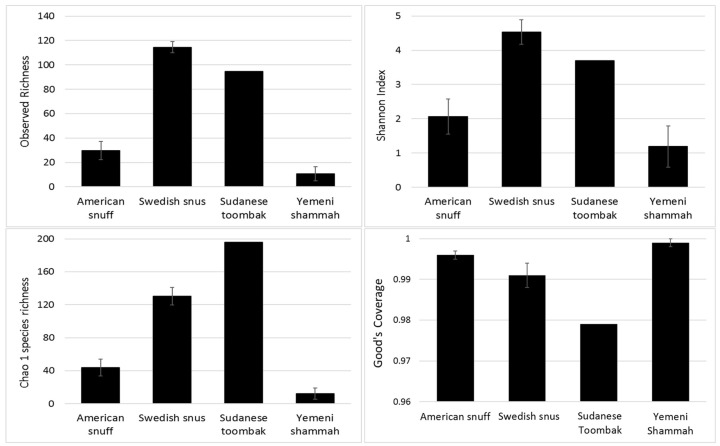
Species richness, α-diversity and coverage (mean ± standard error (SE)) calculated by rarefaction analysis.

**Figure 4:**
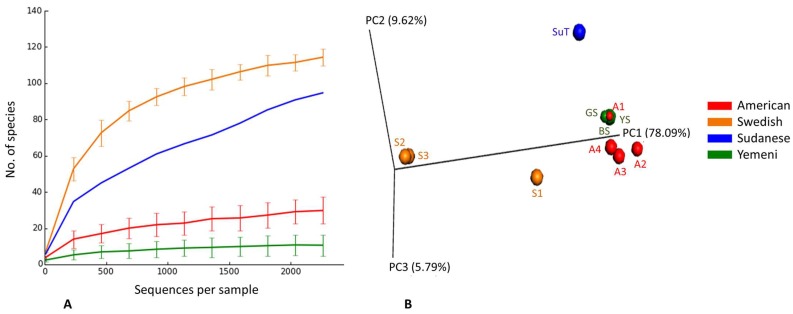
Rarefaction and β-diversity. (**A**) Rarefaction curves showing the number of observed species as a function of sequencing depth; (**B**) Clustering of ST products by principle component analysis (PCoA) based on weighted UniFrac (*p* = 0.01, Analysis of Similarities (ANOSIM)).

The observed and expected number of species (Chao1), Shannon index (α-diversity) and Good’s coverage, calculated by rarefaction based on the sample with lowest read count (2265 reads) are presented in Figure 3 (results for individual samples shown in Supplementary Figure S1). The observed species richness and diversity were highest for the Swedish snus and lowest for the Yemeni Shammah. SuT showed the highest expected number of species (Chao1) but the lowest Good’s coverage, which is consistent with the rarefaction curves (Figure 4A): by extrapolation, an additional 2000 reads would have been required to obtain a coverage of 0.99 (saturation) for SuT. The result of principle component analysis (PCoA) based on weighted UniFrac is presented in Figure 4B. Each tobacco type formed a separate cluster, with the exception of the American A1 that clustered with the Yemeni shammah, and the Swedish S1 that fell between the American and other Swedish products. Grouping was found to be statistically significant by Analysis of Similarities (ANOSIM; *p* = 0.01).

### 3.4. Bacteriome Identified in the ST Products

**Figure 5:**
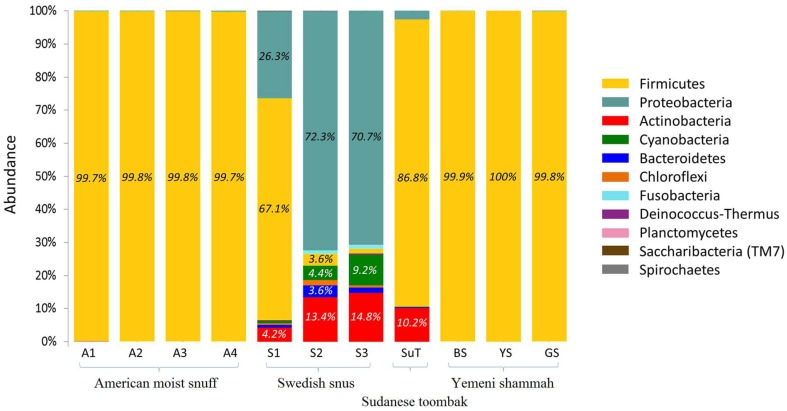
Bacteriome of the ST products—phylum level. Stacked bars showing the relative abundance of bacterial phyla identified in each of the ST products.

A total of 11 phyla were identified in the samples as shown in Figure 5. The number of phyla per sample ranged from one to three for the Yemeni samples, three to five for the American and Sudanese samples, and eight to 10 for the Swedish samples (Supplementary Table S1). Phylum Firmicutes accounted for >99.7% of sequences in all American and Yemeni samples; it also predominated in SuT and S1. Instead, Proteobacteria was the predominant phylum in S2 and S3, and accounted for a significant proportion of the reads in S1. Actinobacteria was identified in all Swedish samples as well as in SuT. S2 and S3 in addition contained considerable levels of Cyanobacteria, Bacteroidetes, Chloroflexi, and Fusobacteria. Using the Bayesian classifier, almost identical results were obtained at the phylum level (See Supplementary Figure S2).

**Figure 6:**
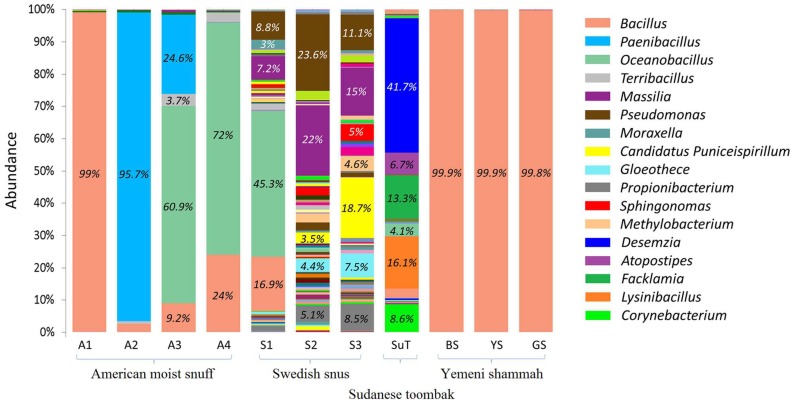
Bacteriome of the ST products—genus level. Stacked bars showing the relative abundance of bacterial genera identified in each of the ST products. Labels and names are shown for genera with relative abundance ≥3%.

The genus-level bacteriome of each of the ST samples is presented in Figure 6. A total of 178 genera were identified, 36 of which were at relative abundance ≥1% (Supplementary Table S2). The number of genera per sample ranged from three to nine for the Yemeni shammah, 10 to 12 for the American snuff, and 78 to 84 for the Swedish snus; 38 were detected in SuT. The genus *Bacillus* constituted >99% of the reads in the Yemeni varieties and American A1, and made up significant proportions in A3, A4 and the Swedish S1. *Paenibacillus* was the predominant genus in A2, while *Oceanobacillus* accounted for the majority of sequences in A3 and A4 as well as S1. The Swedish products S2 and S3 had a totally different profile with a mix of genera *Massilia*, *Pseudomonas*, *Candidatus Puniceispirillum*, *Gloeothece*, *Propionibacterium*, *Sphingonomas*, and *Methylobacterium* making up the bulk of the bacteriome. SuT also had a unique composition with genera *Desemzia*, *Atopostipes*, *Facklamia*, *Lysinibacillus* and *Corynebacterium* accounting for ≈90% of the reads. Using the alternative classification pipeline (Wang’s method) a significant proportion of the reads in some samples were unclassified at the genus level; however, for reads that returned genus-level taxonomies, the results were comparable to that obtained by the BLASTN pipeline (See Supplementary Figure S3).

**Figure 7:**
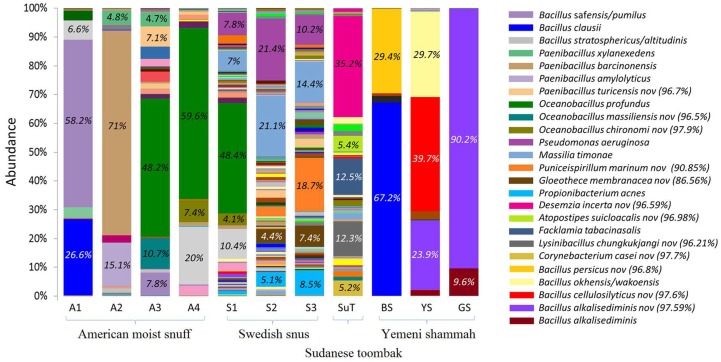
Bacteriome of the ST products—species level. Stacked bars showing the relative abundance of bacterial species identified in each of the ST products. Labels and names are shown for species with relative abundance ≥4%. For potentially novel species, the name of the closest match and % identity is provided.

Figure 7 illustrates the species-level bacteriome of each of the ST products. A total of 491 species-level taxa were identified, of which 66 had a relative abundance of ≥1% (Supplementary Table S3). The number of species per sample ranged from 10 to 23 for the Yemeni shammah, 28 to 43 for the American snuff, and 121 to 139 for the Swedish snus; 95 were detected in SuT. The most abundant species in the American products were *Bacillus* saf*ensis/pumilus* (A1 and A3), *Bacillus stratosphericus/altitudinis* (A1 and A4), *Bacillus clausii* (A1), *Paenibacillus barcinonensis* (A2), and *Oceanobacillus profundus* (A3 and A4). The predominant species in the Swedish varieties S2 and S3 were *Pseudomonas aeruginosa*, *Massilia timonae*, *Propionibacterium acnes* in addition to two potentially novel species with no close relatives: the closest species were *Puniceispirillum marinum* (90.85%) and *Gloeothece membranacea* (86.56%). S1 harbored a mixture of the species found in A4 and those identified in S2 and S3. In SuT, *Facklamia tabacinasalis* in addition to three potentially novel species close to *Desemzia incerta* (96.59%), *Atopostipes suicloacalis* (96.98%)*,* and *Lysinibacillus chungkukjangi* (96.21%) accounted for the majority of the bacteriome. The composition of the bacteriome varied significantly across the Yemeni samples. BS primarily contained *Bacillus clausii* and a novel species close to *Bacillus persicus* (96.8%), while YS harbored *Bacillus okhensis/wakoensis* and two novel species close to *Bacillus cellulosilyticus* (97.6%) and *Bacillus alkalisediminis* (97.59%). The latter novel species made the majority of the bacteriome of GS. To demonstrate reproducibility of sequencing, we carried out comparison of bacterial species profile obtained from two sequencing runs carried one year apart for four of the samples (see Supplementary Figure S4).

### 3.5. Differentially Enriched Microbial Genes and Pathways

**Figure 8:**
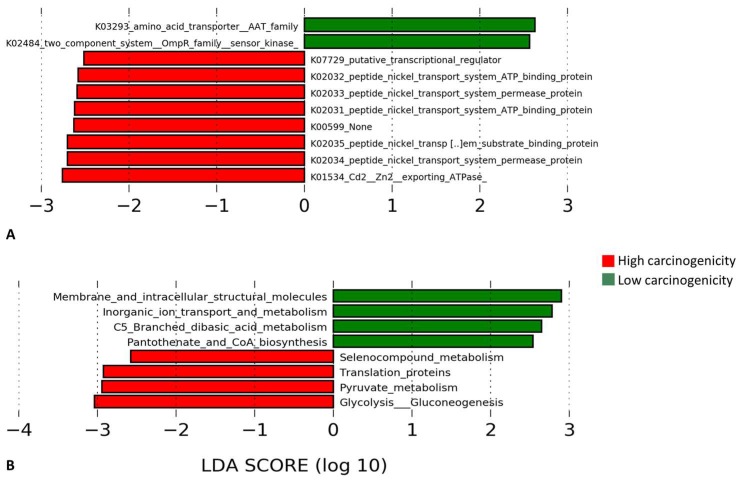
Differentially enriched functions. Linear Discriminant Analysis Effect Size (LEfSe) analysis showing genes (**A**) and pathways (**B**) that were significantly differentially enriched between the low and high carcinogenicity groups (showing those with LDA score ≥2.5).

The microbial genes and pathways enriched in each of the two groups are presented in in Figure 8. At the gene level, genes encoding cadmium/zinc transporting ATPase and peptide nickel transport system permease and ATP binding proteins were enriched in the high carcinogenicity group while those encrypting Amino Acid Transporter (AAT) family and two-component system, OmpR family, sensor kinase were overrepresented in the low carcinogenicity samples. At the pathway level, genes involved in glycolysis/gluconeogenesis, pyruvate metabolism, translation, and selenocompound metabolism were significantly more abundant in the high carcinogenicity group, while those encoding membrane and intracellular structural molecules and involved in inorganic iron transport and metabolism, C5-branched dibasic acid metabolism and pantothenate and CoA biosynthesis were the most significantly overrepresented in the low carcinogenicity products.

## 4. Discussion

The purpose of this study was to elucidate the differences in composition and function of the bacteriome among ready-to-use ST products with different oral carcinogenicity. The ST products were selected so as to include the most commonly consumed brands/types in each of the four countries. In fact, this is the first study to perform microbial profiling of Swedish snus and Yemeni shammah. One limitation, however, is that only one sample per product was examined, missing the opportunity to assess variation in microbial composition across batches. Another limitation is that there is a possibility that part of the microbiome in the samples was not captured because DNA extraction was performed on a supernatant rather than the solid material; however, the idea was to avoid high plant DNA background in the extracts. The SuT had lower coverage than other samples, i.e., did not reach saturation, but was still included in the analysis. Although rarefaction is a common practice in microbiome studies, there is some evidence to suggest that it may result in loss of power and should thus not be performed [40]. In the current study, however, rarefaction was used only to normalize counts for calculation of coverage and diversity indices; the complete microbiome was employed in assessing differential abundance and performing the functional analysis.

By sequencing the V1–V3 region with Illumina’s 2 × 300 paired-end chemistry and merging the resultant reads, relatively long reads (472–562 bp) were generated which improved taxonomic resolution. Using a BLASTN-based algorithm inspired from previous work [32], we ventured to classify the reads to the species level. Very stringent read quality-filtration was implemented which, while negatively impacted on sequencing depth by eliminating the majority of reads (another study limitation), ensured the lowest possible sequencing errors rate and in turn maximized reliability of the species-level assignment. Nevertheless, the results of classification should be interpreted with caution. The reference databases used comprise mainly 16S rRNA sequences of named species. Therefore, a species to which a read is assigned may not necessarily be the same species from which the read was obtained, but rather the closest named species to it. Despite this limitation, this is probably more informative than limiting classification to higher ranks. For comparison purposes, we also classified the reads using Wang’s method (a Bayesian classifier) against Greengene version 13.5 reference database. While the taxonomy assignments were almost identical at the phylum level to that obtained by our pipeline and a significant proportion of the reads were not classified at the genus level, which is probably why the results reported by Tyx et al. [21] were limited to the family level.

As expected, the Swedish snus harbored far lower bacterial counts than the other products, which is consistent with the fact that Swedish snus products are subject to pasteurization during their manufacturing process, and also explains the low levels of TSNAs present in them [41]. A considerable proportion of the quantified DNA in the Swedish sample possibly represented non-viable bacteria. The Yemeni YS had the highest microbial load. The levels of TSNAs in Yemeni shammah have not been yet established, but can be assumed to be high in view of the strong association between shammah use and oral cancer [8,10]. The American, Sudanese, and Yemeni products had comparable bacterial loads. However, studies indicate that they significantly vary with respect to their TSNA content [42]; SuT in particular has very high concentrations of TSNAs [43]. Obviously then, the levels of TSNAs in ST products differ not only as function of total microbial load but probably also as a function of microbial community composition. Indeed, a very recent study has found the TSNA concentrations in tobacco leaves to correlate positively with the proportions of Firmicutes and inversely with those of Proteobacteria [20]. Interestingly, Firmicutes was the predominant phylum in the American, Sudanese, and Yemeni products in this study, while Proteobacteria was the major phylum in the Swedish products.

Tyx et al. [21] recently reported on the microbiology of American moist snuff and Sudanese toombak using sequencing of the hypervariable region V4. Although description of the results was limited to the family level, the supplementary material did provide genus-level information that can be directly compared with our results. In their study, *Tetragenococcus*, *Aerococcus*, *Alliococcus*, *Staphylococcus*, and an unclassified genus of the family *Aerococcaceae* were identified as the predominant genera in the American moist snuff, which is markedly inconsistent with the current study in which *Paenibacillus*, *Oceanobacillus*, and *Bacillus* were the most abundant genera in the corresponding samples. *Desemzia*, *Facklamia*, *Lysinibacillus*, and *Atopostipes*, four out of the five most abundant genera in SuT in the present study, were either not detected or detected at very low abundance in their SuT samples, in which *Corynebacterium*, *Staphylococcus*, and an unclassified genus of the family Aerococcaceae made up the bulk of the microbiome instead. While these vast differences in results between the two studies may be explained in part by methodological variations, they probably reflect genuine differences in the microbial composition of samples used in the two studies. Since the products were coded in both studies, it is very likely that the two studies included different brands of American moist snuff. SuT is produced widely across Sudan in non-standard production settings, so its microbiological composition can be expected to vary significantly as a function of where and how it is produced. Obviously, a larger-scale study including representative samples of each ST product would be necessary to resolve this question.

Despite having the lowest bacterial load, the Swedish snus displayed the highest diversity at all taxonomic levels. With the exception of S1, which looked like a blend of A4 and S2/S3, the Swedish products were almost free of the phylum Firmicutes; a significant proportion of the sequences belonged to human-associated taxa, namely *M.* *timonae*, *P. aeruginosa*, and *P. acnes*, which probably represent contamination at a later stage of production. Interestingly, the presence of genus *Pseudomonas* in tobacco has been shown very recently to inversely correlate with TNSAs levels [20]. Novel, probably environmental, taxa also accounted for a considerable fraction of the reads in the Swedish products. The Yemeni shammah showed the lowest diversity, with almost all of the reads belonging to known and novel species of the genus *Bacillus*. The majority of the reads in SuT also represented novel species. Isolation and characterization of the novel species from Yemeni shammah and SuT is, therefore, warranted not only to ascertain their ability to accumulate nitrites, but also to assess their direct effects on oral epithelium.

Differences in predicted functions between the presumptively low carcinogenicity and high carcinogenicity products at the pathway level did not seem to be relevant to oral carcinogenesis. However, the differences at the gene level did. Genes encoding cadmium/zinc and nickel transport systems were enriched in the presumptively “high carcinogenicity” products, suggesting these heavy metals are present at higher concentrations in them. Cadmium is considered as a carcinogen by the International Association on Cancer Research (IACR) [44]. Interestingly, it is also present in ST products [45], although its role in oral carcinogenicity has not been assessed. Nickel is linked in the literature to nasal and lung cancers [46]; recently, it has also been implicated in oral cancer [47,48]. In addition, it has been detected in ST products [49]. Together, this suggests, keeping in mind this is only based on predictive functional analysis, that cadmium and nickel may be important carcinogens in Yemeni and Sudanese ST products.

A microbial community with high nitrate reducing but low nitrite reducing properties (incomplete denitrification) is probably required to support formation of high levels of TSNAs in tobacco [13]. Predictive metagemonic analysis (PICRUSt), however, did not show a significant difference in abundance of nitrate or nitrite reductase genes between the low and high carcinogenicity product groups, although, the formate- dependent and NO forming nitrite reductase genes tended to be enriched in the Swedish snus samples (data not shown). In fact, production of TSNA is not only dependent on ability of bacteria to accumulate nitrites but also on environmental factors e.g., moisture, temperature, pH, the nitrite/nitrate content of the product, etc. [1,50]. In any case, since TSNA levels were not directly measured in the samples included in this study, the attempts made here to correlate between the bacterial composition and the assumed TSNA concentrations based on the literature are at best speculative.

## 5. Conclusions

The current study demonstrates that ST products differ qualitatively, quantitatively, and functionally in their bacterial composition. However, a larger-scale study involving more representative samples of each type is required to uncover the full breadth of microbial diversity across these products. The high taxonomic resolution used here helped identify the reads to the closest species; several potentially novel species were identified. The possibility that some of these species contribute to oral carcinogenesis, either via influencing levels of TSNAs or directly inducing chronic inflammation, warrants further investigation. Imputed functional prediction did not demonstrate a difference in potential for TSNA production between low and high carcinogenicity products; however, it did suggest that the presumptively high carcinogenicity products have higher concentrations of nickel and cadmium; this needs to be confirmed using whole metagenome sequencing as well as chemical analysis.

## Supplementary Materials

The following are available online at www.mdpi.com/2073-4425/8/4/106/s1. Table S1: List of all phyla identified and their abundance in each of the ST products. Table S2: List of all genera identified and their abundance in each of the ST products. Table S3: List of all species-level taxa identified and their abundance in each of the ST products. Figure S1: Species richness, α-diversity and coverage for the individual samples. Figure S2: The bacteriome profile at the phylum level obtained using the Bayesian classifier and Greengene version 13.5 as reference. Figure S3: The bacteriome profile at the genus level obtained using the Bayesian classifier and Greengene version 13.5 as reference. Figure S4: Reproducibility of sequencing. Comparison of results from two sequencing runs, one year apart, for 4 of the ST products.
