# Supplementary figures and images for: Differences in the Bacteriome of Smokeless Tobacco Products with Different Oral Carcinogenicity: Compositional and Predicted Functional Analysis
Source: Genes (Basel). 2017 Mar 23;8(4):106. doi: 10.3390/genes8040106 (PMC5406853; doi:10.3390/genes8040106)

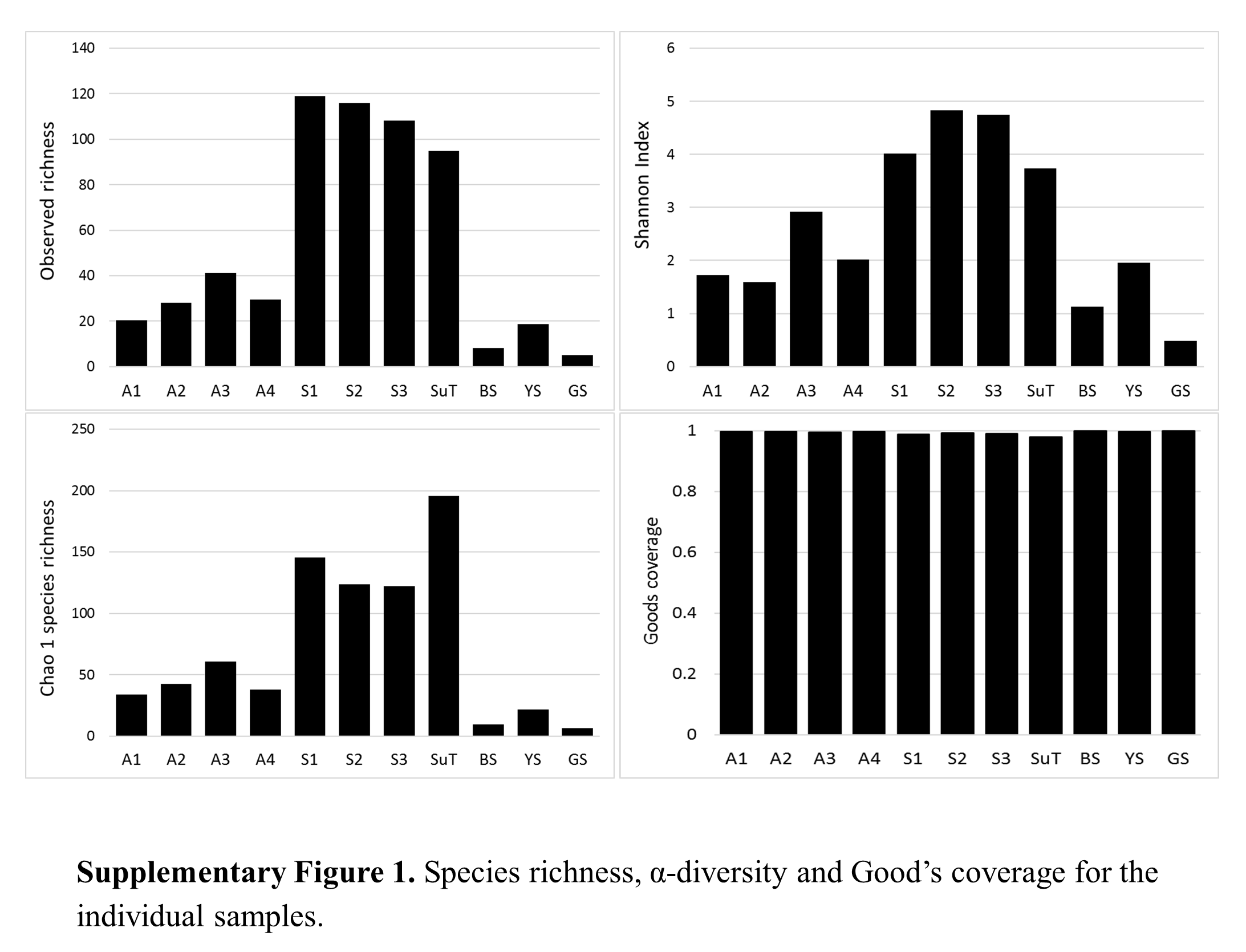

Supplement: Supplementary file 1 [file genes-08-00106-s001.zip › Figure S1.tif]

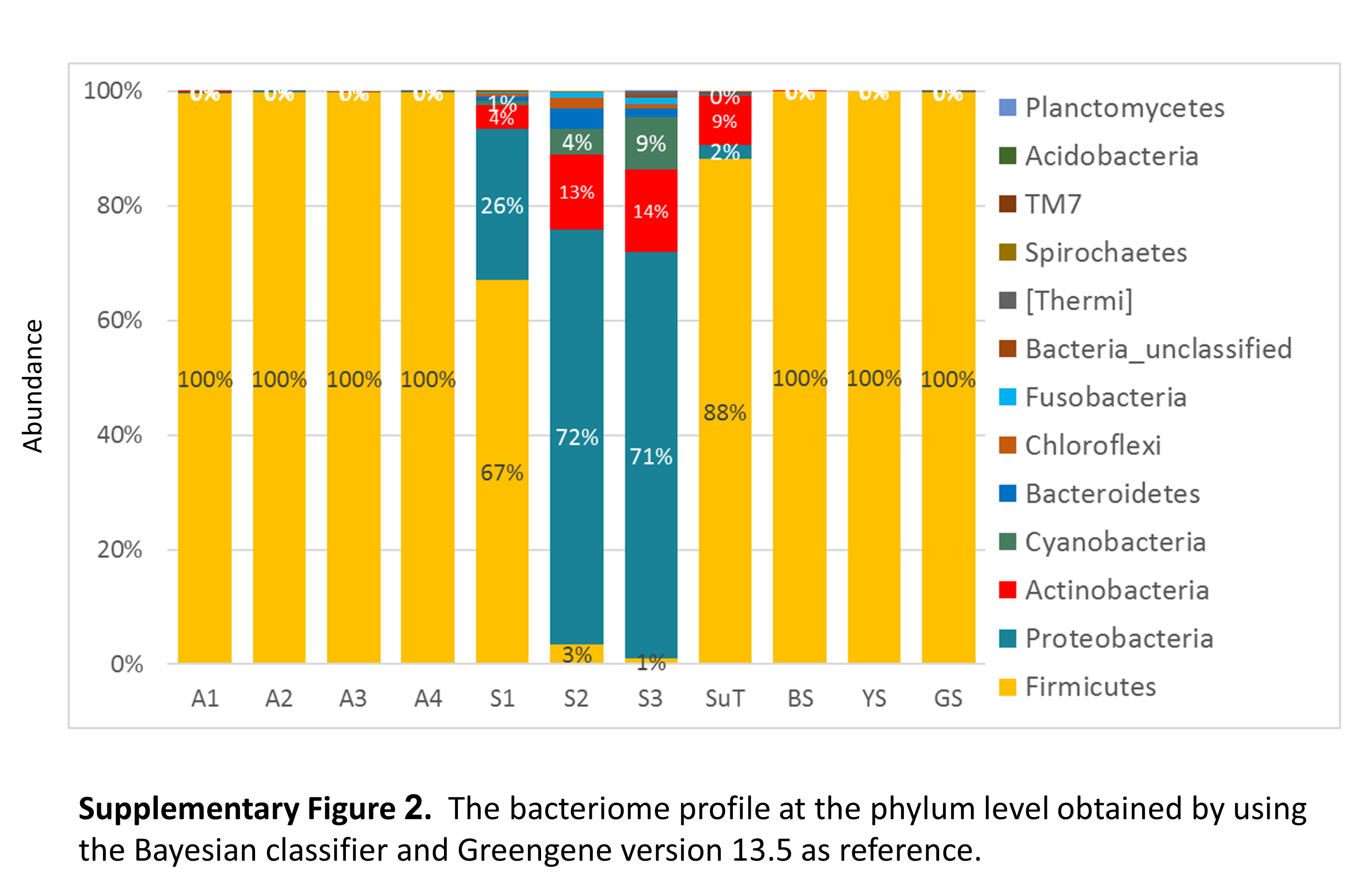

Supplement: Supplementary file 1 [file genes-08-00106-s001.zip › Figure S2.tif]

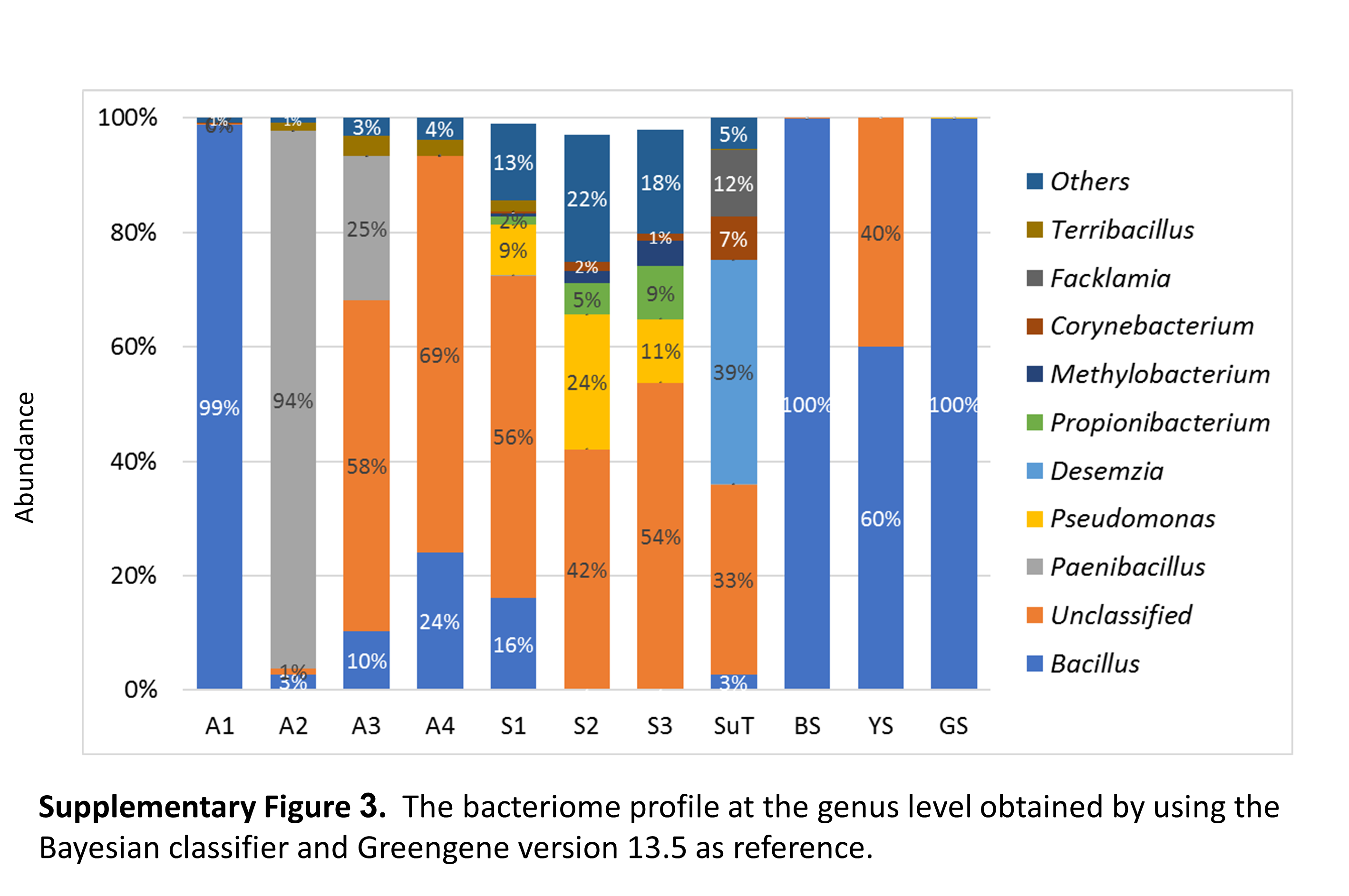

Supplement: Supplementary file 1 [file genes-08-00106-s001.zip › Figure S3.tif]

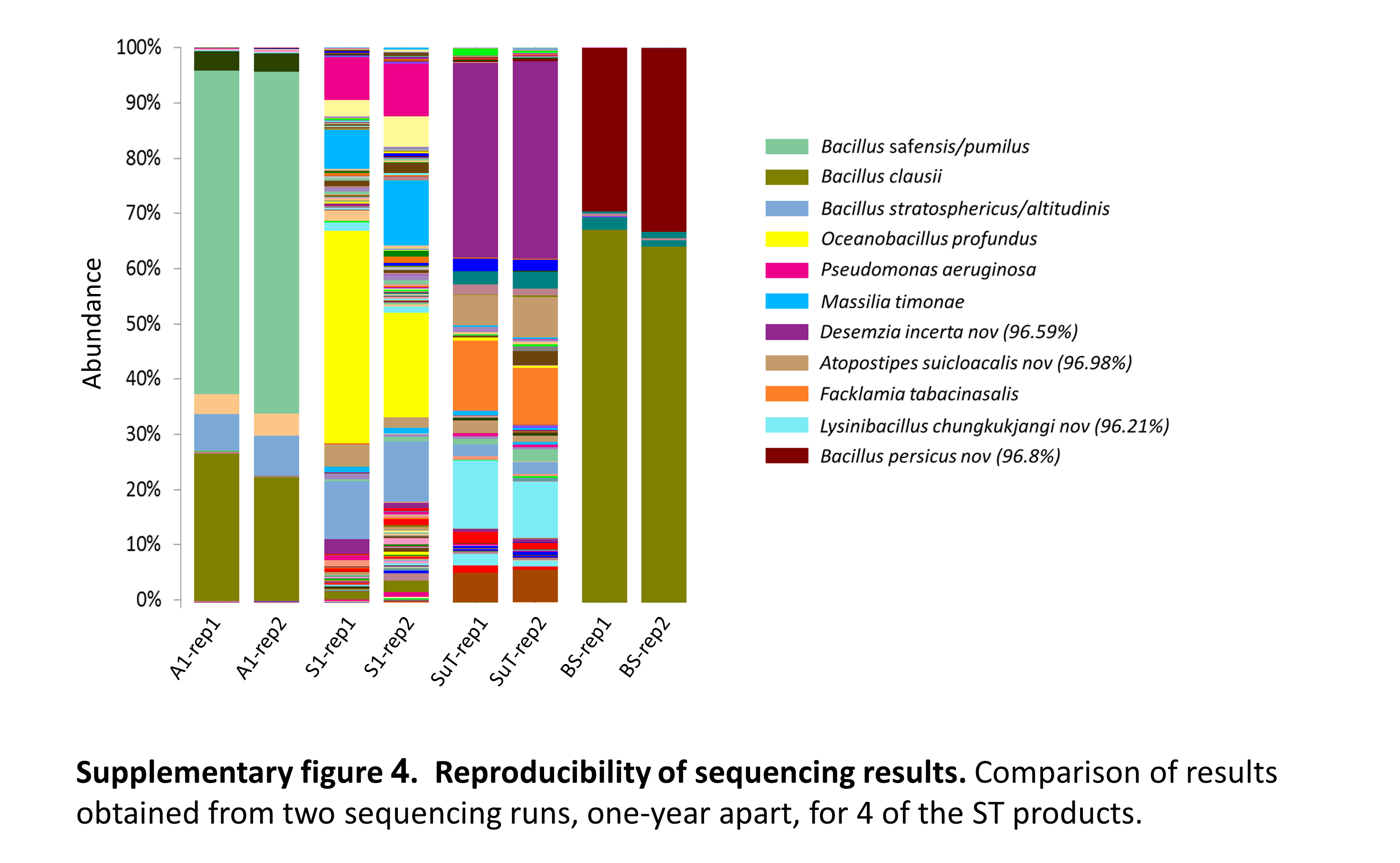

Supplement: Supplementary file 1 [file genes-08-00106-s001.zip › Figure S4.tif]
